# H2A.Z Nucleosome Positioning Has No Impact on Genetic Variation in *Drosophila* Genome

**DOI:** 10.1371/journal.pone.0058295

**Published:** 2013-03-05

**Authors:** Yitao Tang, Shan Dong, Xinkai Cao, Qing Zhou, Guitao Ding, Cizhong Jiang

**Affiliations:** Shanghai Tenth People’s Hospital, Department of Bioinformatics, Shanghai Key Laboratory of Signaling and Disease Research, The School of Life Sciences and Technology, Tongji University, Shanghai, China; University of California, Davis, United States of America

## Abstract

Nucleosome occupancy results in complex sequence variation rate heterogeneity by either increasing mutation rate or inhibiting DNA repair in yeast, fish, and human. H2A.Z nucleosome is extensively involved in gene transcription activation and regulation. To test whether H2A.Z nucleosome has the similar impact on sequence variability in the *Drosophila* genome, we profiled the H2A.Z nucleosome occupancy and sequence variation rate at gene ends and splicing sites. Consistent with previous studies, H2A.Z nucleosome positioning helps to demarcate the borders of exons. Nucleosome occupancy is anticorrelated with sequence divergence rate in the regions flanking transcription start sites and splicing sites. However, there is no rate heterogeneity between the linker DNA and H2A.Z nucleosomal DNA regardless of nucleosome occupancy, fuzziness, positioning in promoter, coding, and intergenic regions, young or old genes. But the rate at intergenic nucleosomes and the flanking linker regions is higher than that at the genic counterparts. Further analyses found that the high sequence divergence rate in the promoter regions that are usually nucleosome depleted regions may be likely resulted from the high mutation rate in the enriched tandem repeats. Interestingly, within nucleosomes spanning splicing sites, sequence variability of nucleosomal DNA significantly increases from the end within exons to the other end protruding into introns. The relaxed functional constraint in introns contributes to the high rate of nucleosomal DNA residing in introns while the strict functional constraint in exons maintains the low rate of nucleosomal DNA residing in exons. Taken together, H2A.Z nucleosome occupancy has no effect on sequence variability of *Drosophila* genome, which is likely determined by local sequence composition and the concomitant selection pressure.

## Introduction

Nucleosome is the fundamental unit of chromatin in eukaryotes, consisting of approximately 147 bp of DNA coiling around a histone octamer [1], [2]. Nucleosome positioning has been shown to be involved in gene transcription [3], demarcation of exon borders [4], [5], mRNA splicing [6], DNA replication [7], and DNA repair [8]. Nucleosomes exert these functions mainly through regulating the accessibility of DNA. DNA sequence composition has an impact on nucleosome positioning. For example, the rigid poly (dA:dT) tracts in the promoters of yeast genes disfavors nucleosome formation and results in a nucleosome free region [9], [10], [11], [12]. Therefore, mutations in genomic regions can change the ability of DNA sequence to bend and alter nucleosome positioning as a result. Conversely, nucleosome occupancy can affect the efficiency of DNA repair by generating a barrier for repair enzymes or change mutation rate by protecting the sequence from damaging agents [13], [14]. Therefore, there is likely a co-evolution of genomic DNA sequence conservation and chromatin structure.

With the advances in high-throughput sequencing technology, the genome-wide maps of mononucleosome positions have been produced in model organisms [15], [16], [17], [18]. These maps made it possible to investigate the interplay between sequence variability and nucleosome positioning. A ∼10% lower substitution rate was observed in the linker regions than in nucleosomal DNA in yeast genome [19]. Interestingly, genetic variation downstream of transcription start sties (TSSs) showed a distinctive ∼200-bp periodicity correlated with nucleosome positioning in medaka (Japanese killifish) genome. The substitution rate increased toward the dyad (the equivalent of nucleosome center) in promoter regions. Contrary to this, the indels were more likely to occur in the linker regions [20]. Similarly, indels were depleted in the DNA sequences occupied by the bulk and epigenetic nucleosomes (H2A.Z and H3K4me3) in the human genome. However, single-nucleotide polymorphisms (SNPs), behaving more intricately, were enriched on bulk nucleosomes but depleted on the epigenetically modified nucleosomes when compared to the linker regions [21]. Another recent study found that local GC content played a critical role in selection on nucleosomal DNA and the neighboring linker DNA sequences. The optimum GC composition in core and linker regions was maintained by the elevated rate of C→T substitutions in the linker regions and decreased within nucleosome core regions [22]. Taken together, these findings implied a complex interplay between genomic sequence variability and nucleosome positioning.

In order to test whether H2A.Z nucleosomes have similar impact on genetic variation in *Drosophila* genome and to what extent of the impact, we collected substitutions and indels from the *Drosophila* multiple-species alignment data set available at UCSC Genome Bioinformatics sites. The H2A.Z nucleosome data was from our previous work [16]. We explored the interplay between sequence variation and nucleosome position at TSSs, transcription terminal sites (TTSs), splicing sites and intergenic regions. The results reveal negatively correlated profiles of sequence variability and H2A.Z nucleosome occupancy around TSSs, TTSs, and splicing sites. Substitutions and indels occur more frequently in the promoter regions where H2A.Z is depleted, and less frequently in the genic regions downstream of TSSs where H2A.Z is enriched. The high sequence variability in the promoters is very likely attributed to the enriched tandem repeats whose mutation rate is high. Intriguingly, we did not observe H2A.Z nucleosome-related rate heterogeneity. The sequence divergence rate is similar between the linker regions and nucleosomal DNA regardless of H2A.Z genomic locations. Further analyses implied that H2A.Z had no impact on sequence variability, which was mainly determined by the local sequence composition.

## Materials and Methods

### Nucleosome Data and Gene Annotation

H2A.Z nucleosome sequencing data was from our previous work [16]. The coordinates of transcripts, exons, and introns were downloaded from Flybase (release 5.30).

### Genome Sequence Variation Data

The *Drosophila* multiple-species alignment data set was downloaded from UCSC Genome Bioinformatics sites (version: Apr. 2006, BDGP R5/dm3). The inferred phylogeny of the 12 *Drosophila* species showed a clade consisting of *D. melanogaster, D. simulans,* and *D. sechellia* [23]. This clade included both cosmopolitan species (*D. melanogaster, D. simulans*) and the isolated species that live on single islands (*D. sechellia*). Moreover, the clade had strong support from both Bayesian and maximum parsimony. Therefore, the alignment of these three species could provide a robust source for sequence divergence on a genomic scale. We defined substitutions at the sites where the nucleotides are identical in *D. simulans,* and *D. sechellia* but different from *D. melanogaster*. The indels included two types of sites: one, there is a gap only in *D. melanogaster*; two, there is a nucleotide only in *D. melanogaster* and a gap in both *D. simulans* and *D. sechellia*. In the end, we obtained a total of 6,709,569 substitutions and 2,905,425 indels.

### Nucleosome and Sequence Variation Distribution Profiles at Gene Ends

The genome positions of nucleosome sequencing reads were retrieved from our previous work [16]. Briefly, we shifted 5′ end position of each read 73 bp toward 3′ end as the position of the nucleosome midpoint. The nucleosome midpoints located in the effective regions of TSSs were collected. The 5′ border of the effective region was the closer of 1 kb and the end of the adjacent gene. The 3′ border was the closest of 1 kb, its own TTS, and the end of the adjacent gene. The nucleosome density in a certain distance to 5′-most TSS of all genes was calculated and binned in 5-bp interval. Bin data were normalized to number of genes represented in each bin, and smoothed using a moving average of five bins with a step window of one bin. Thus, we obtained the nucleosome distribution profile around TSS. The reason to use 5′-most TSS of each gene was to eliminate TSSs located internal to genes and remove redundancy due to alternative transcripts. However, we obtained the indistinguishable nucleosome distribution profile when we used the 3′-most TSS for each gene or all TSSs (data not shown).

The nucleosome distribution profile around TTSs was analyzed using the similar method except that 3′-most TTS of all genes were used. Similarly, the sequence variation rate profiles at both gene ends were analyzed as the nucleosome distribution profiles.

### Nucleosome and Sequence Variation Distribution Profiles at Splicing Sites

We first defined internal exons as the ones that were embraced by an intron at both ends. The redundant exons from alternative transcripts were removed. We retained 28,640 internal exons. The 5′ and 3′ end of internal exons are acceptor and donor sites, respectively. The effective region of a splicing site is defined by two borders: one is the smaller of 1 kb and intron length to the splicing site; the other is the smaller of 1 kb and exon length. Nucleosomes located in the effective regions of splicing sites were collected. The nucleosome density in a certain distance to splicing sites was calculated and binned in 5-bp interval. Bin data were normalized to number of regions represented in each bin, and smoothed using a moving average of five bins with a step window of one bin. The sequence variation rate profiles at splicing sites were calculated in a similar manner.

### Sequence Variation Distribution at the Linkers and Nucleosomes

The coordinates of H2A.Z nucleosomes predicted from sequencing data were from our previous work [16]. The sequence variations on the linkers and nucleosomal DNA were collected for sequence variation rate calculation.

## Results

### Negative Correlation between Genetic Variation Frequency and H2A.Z Nucleosome Occupancy at Both Gene Ends and Splicing Sites

**Figure 1:**
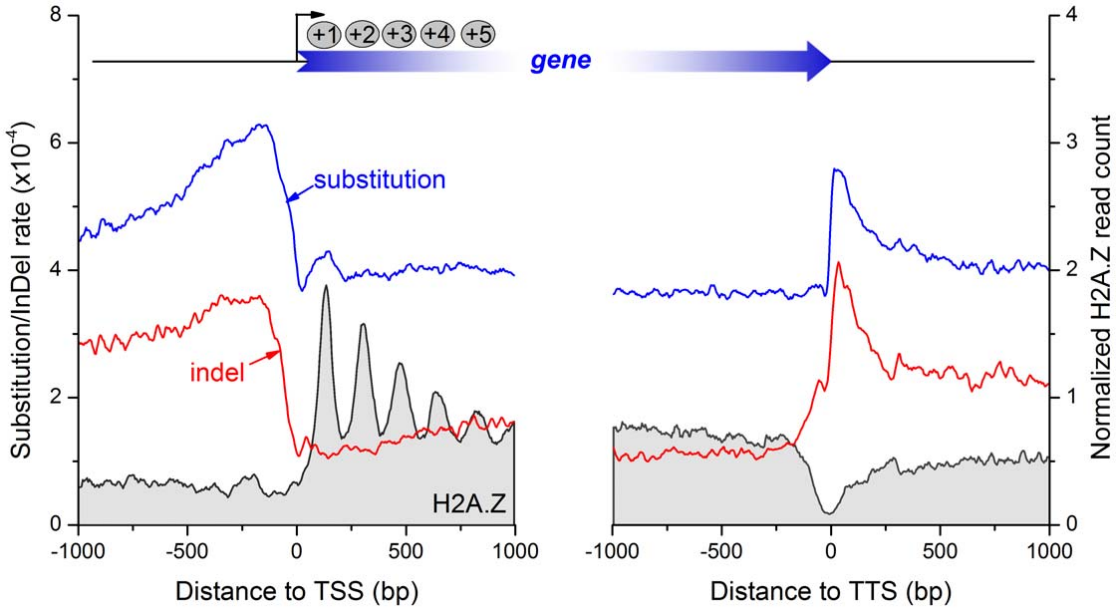
Negative correlation between sequence divergence rates and nucleosome occupancy around gene ends. The left panel shows the distribution of substitution rate (blue), indel rate (red), and H2A.Z nucleosome occupancy (gray area) around gene start site. H2A.Z is enriched in coding region by a canonical +1, +2, etc. uniform nucleosome organization, and depleted in the promoter region. The top mega gene indicates positioning of the first five nucleosomes downstream to TSS. In contrast, both substitution and indel rates are much higher in the promoter region than in coding regions, and peak at ∼200 bp upstream of TSS. The right panel shows the situation around gene end site. H2A.Z occupancy is lower in gene end site than in gene start site, and lacks a uniform positioning. A ∼200-bp nucleosome depleted region centers at TTS. Substitution and indel rates are low at gene end, but precipitously increase immediately after TTS.

The multiple alignments with *D. melanogaster* from UCSC genome database have provided a robust resource to identify genetic variations in *D. melanogaster*. We collected substitutions and indels in *D. melanogaster* from the multiple alignments (see Methods for details). Both substitutions and indels are enriched in the promoter region peaking at 200 bp upstream of TSS, and are depleted in the coding region downstream of TSS (Fig. 1). The indel dataset was then split into 1-bp and >1-bp two groups. Both 1-bp and >1-bp indels show the similar distribution pattern (Fig. S1). In contrast, H2A.Z nucleosomes are depleted in the promoter regions and are enriched in the genic regions downstream of TSS with a canonical uniform distribution. The open chromatin at 5′ end of genes is important for the assembly of transcription machinery. The 5′ nucleosome depleted regions (NDRs) might also allow low basal levels of leaky transcription and maintain the constitutive low expression of genes with a housekeeping function [3]. Both substitutions and indels have a valley at the TSS, suggesting functional conservation and selection constraint at the TSS. Unlike in medaka [20], we did not see the ∼200-bp periodicity of the substitution and indel rates in the coding region. However, genome variation and H2A.Z nucleosome profiles overall negatively correlate at both the promoter and coding regions (Fig. 1). This is consistent with the previous observations in medaka [20] and human [21].

Sequence variations remain at a stable low level in the downstream coding region, increase precipitously immediately after the 3′ end of genes and drop at the further downstream intergenic region (Fig. 1). The H2A.Z nucleosome level is globally low around the 3′ end of genes with an NDR at TTS. The NDR may facilitate the nascent transcript to get off DNA template and yield space for the next transcript [3]. Interestingly, indel frequency is negatively correlated with nucleosome occupancy around TTS in human, whereas SNP frequency is positively correlated [21]. Consistent with the earlier finding of indel, but contrary to the positive correlation of substitution found in human, both substitution and indel frequency is negatively correlated with H2A.Z nucleosome occupancy around TTS in *Drosophila*, albeit the peak of genome variations spans only half of NDR (Fig. 1).

**Figure 2:**
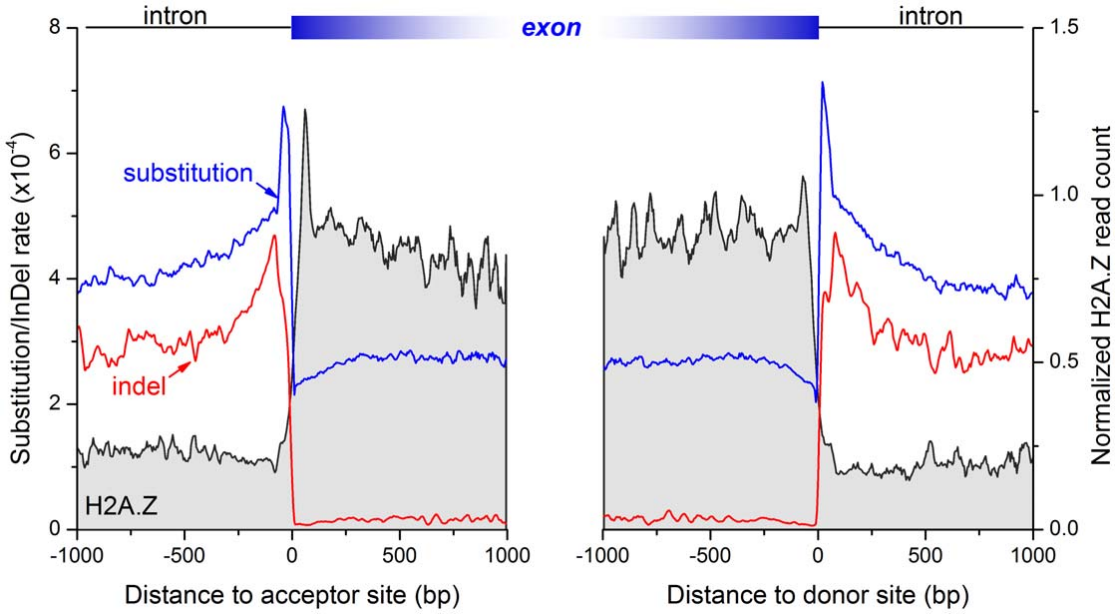
Inverse association between sequence divergence rates and nucleosome occupancy around splicing sites. The left panel shows genetic variation rates (blue: substitution, red: indel) and H2A.Z nucleosome occupancy (gray area) around acceptor sites. The right panel shows the case around donor sites. H2A.Z is enriched within exons with a well-positioned nucleosome at both borders, and largely depleted inside introns. Contrary to this, sequence variation rates are very low within exons and precipitously increase inside introns.

Splicing sites are one of the most conserved genomic regions. The dinucleotides at the border of introns at the donor (GT) site and the acceptor (AG) site are the hallmark of intron-exon splitting site and least tolerant with mutation. Compared with introns, exons encoding proteins or ncRNAs have more functional constraints and allow less mutations. Indeed, substitution and indel rates are much higher in introns than in exons, and reach minima at the splicing sites with a prominent peak at the intronic side ∼40 bp to the splicing site. Conversely, H2A.Z nucleosome density is more than 4 fold in exons than introns. Both the borders of exons are embraced with a positioned nucleosome (Fig. 2). The anti-phase of H2A.Z nucleosome positioning and sequence variation frequency around the splicing sites is consistent with the previous findings in human [21].

### H2A.Z Nucleosome Positioning in the Demarcation of Exons

Previous studies have found that the bulk nucleosome organization helped to define exons in several species [4], [5]. H2A.Z is a hallmark of active gene transcription [24] and has an important role in gene transcription. The enrichment of H2A.Z nucleosome in exons and the depletion in introns (Fig. 2) indicate the similar function of H2A.Z in definition of exons. To further confirm this hypothesis, we classified exons by its length. Group I exons of 100–200 bp can hold only one nucleosome, group II exons of 250–300 bp hold two nucleosomes, and group III exons of 450–500****bp hold three nucleosomes. Consequently, we observed one, two, and two-to-three nucleosomes in the exons of group I, II, III, respectively (Fig. S2). The nucleosome at the exon border is more fixed than those inside of exons. The nucleosome is depleted in the introns irrespective of exon length. Moreover, the enrichment and depletion of nucleosome exactly split at the splicing site. Coincidentally, the size of *Drosophila* exons has a peak at approximately 150 bp, roughly the length of nucleosomal DNA (Fig. S3). Taken together, H2A.Z nucleosomes are positioned in a manner to help define exons in *Drosophila* genome.

### No Rate Heterogeneity between Linker Regions and Nucleosomal DNA

**Figure 3:**
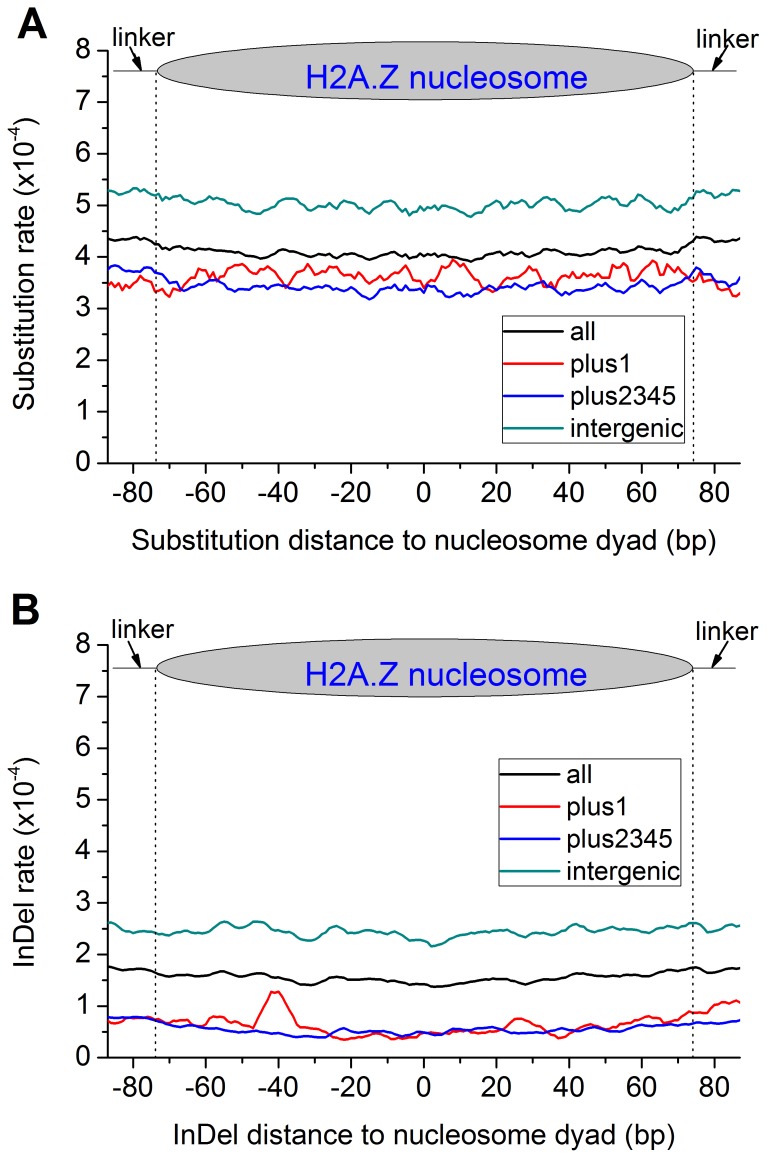
No rate heterogeneity between nucleosomal DNA and linker regions. There is no significant difference in substitution (A) and indel (B) rate between linkers and nucleosomal DNA. All: all nucleosomes, plus1: only +1 nucleosome; plus2345: +2∼+5 nucleosomes representing genic regions; intergenic: nucleosomes in intergenic regions. However, the rates in intergenic nucleosomes are much higher than those in genic regions. The vertical dotted lines indicate the border between linkers and nucleosomes.

The observed differences in genome variation rate between promoter and coding regions, between introns and exons, and the negative correlation between the genome variation rate and H2A.Z nucleosome occupancy in these regions raise the question as whether nucleosome positioning is a causal factor for the rate heterogeneity. To address this question, we examined the substitution and indel rate in linker regions and nucleosomal DNA. Intriguingly, there is no difference in the substitution and indel rate between genome-wide linker regions and nucleosomal DNA (Fig. 3). Previous studies have shown that +1 nucleosome has an important role in gene transcription regulation [15], [16], [17]. We specifically collected +1 nucleosomes and profiled sequence variations along the +1 nucleosomal DNA and the linkers. There is no rate heterogeneity between +1 nucleosomes and the linkers either. We further classified genic nucleosomes (+2, +3, +4, +5 nucleosomes) and intergenic nucleosomes located in intergenic regions. The results turned out no rate heterogeneity between the linkers and the nucleosomes. However, the sequence variation rate is higher in intergenic nucleosomes than in exonic ones. This is likely due to the strict functional constraints in exonic DNA sequences.

Occupancy and fuzziness are two important properties of nucleosomes, measuring nucleosome positioning dynamics in two different perspectives. Occupancy is a quantifier of nucleosome density. The nucleosome occupancy is zero in the regions without any nucleosome binding. Fuzziness measures how fixed the position of the same nucleosome is at different stages or cell states. Nucleosomes are phased whose position changes very little or none at different cell states. Opposite to this are delocalized or fuzzy nucleosomes. We classified four types of nucleosomes: high/low occupancy, phased/delocalized. Our results find that genetic variation rates appear homogenous in the flanking linkers and the nucleosomes of each type (Fig. S4). The overall higher mutation rate in delocalized nucleosomes than phased nucleosomes is in part due to higher portion (38%) of delocalized nucleosomes locating in intergenic regions when compared with phased nucleosomes (29%). This further suggests that sequence composition has more pronounced impact on sequence variability.

### Sequence Composition not Nucleosome Occupancy Leading to Rate Heterogeneity

**Figure 4:**
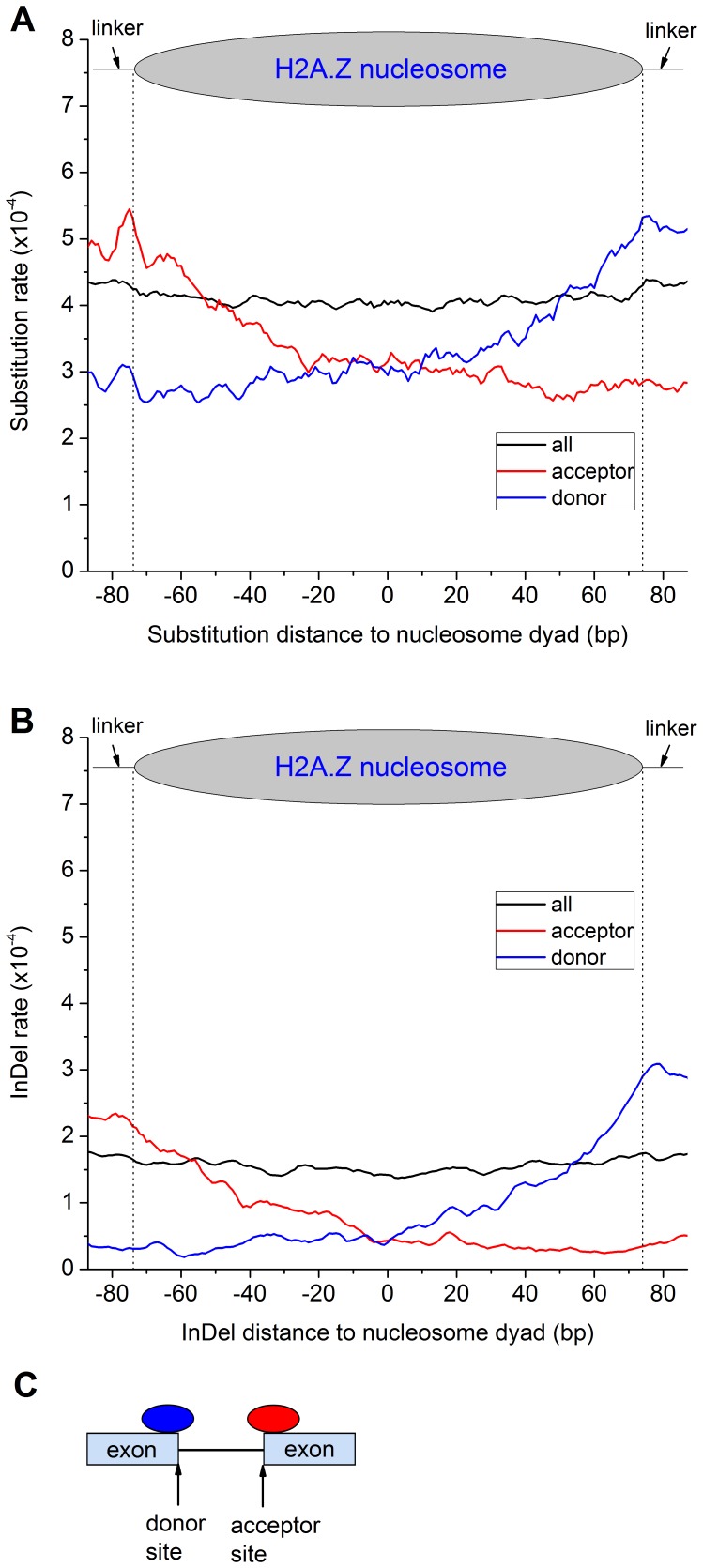
Rate heterogeneity of nucleosomes spanning splicing sites. The substitution (A) and indel (B) rates increase from the exonic end of nucleosomes to intronic sites as nucleosomes protrude into introns, and reach the maximum at the linker regions within introns. The vertical dotted lines indicate the border line between linkers and nucleosomes. C, When nucleosomes spanning the splicing site, the border extrudes into intronic regions. The higher sequence variability in introns than in exons results in the rate heterogeneity in A and B.

As described above, no rate heterogeneity was observed between the linkers and nucleosomal DNA for all and classified nucleosomes (Fig. 3). However, the sequence variation rate is inversely correlated with nucleosome occupancy around both gene ends and splicing sites (Fig. 1 & 2). Additionally, the sequence variation rate in the intergenic linkers and nucleosomal DNA is higher than that in the coding counterparts (Fig. 3). All these together imply that sequence composition not nucleosome occupancy may be the determinant factor in rate heterogeneity. To address this question, we collected nucleosomes spanning splicing sites and examined the sequence variation in the linkers and nucleosomal DNA. One side of these nucleosomes locates in intronic sites and the rest is still in exons. Therefore, these nucleosomes are perfect for testing the interplay of sequence composition and nucleosome occupancy in affecting sequence variation. Intriguingly, both substitution and indel rates increase toward intronic sites as nucleosomes protrude into introns (Fig. 4). Surprisingly, when we used a subset of these nucleosomes requiring that their midpoints locate within exons and at 40–60 bp to the splicing site, thus the nucleosomal DNA within introns and nearest to the nucleosome midpoint is 40 bp. Indeed, both substitution and indel rates of the nucleosomal DNA largely increase starting at 40 bp to the dyad and toward the nucleosome border within intronic site (Fig. S5). This is exactly where the nucleosomal DNA nearest to the dyad starts to extend into intronic sites. The slight rate increase in the other border of nucleosomes may be likely because some nucleosomes protrude into introns, especially for the short exons. Due to higher sequence variation rate in introns than in exons, the rate increases in the nucleosomal DNA sequences extruding into introns. Therefore, sequence composition not nucleosome occupancy is the determinant factor in rate heterogeneity.

The genomes are not uniformly prone to change because of different local sequence composition. Tandem repeats (TRs) are one of the hotspots for mutating events, and mutate 100 to 10,000 times faster than point mutations [25]. We searched all repeats in *Drosophila* genome using the tool Tandem Repeat Finder (Version 4.04) [26]. and found the enrichment of TRs in promoters (Fig. S6). This is consistent with the previous study in yeast genome [27]. Interestingly, TRs peak around 200 upstream of TSS where substitution and indel peak. TRs are depleted at TSS. Therefore, it’s likely that the high mutation rate in TRs contribute to the high sequence variation in the promoters.

## Discussion

Our study examined the interplay between H2A.Z nucleosome positioning and sequence variability in *Drosophila* genome. Like bulk nucleosomes in other organisms, H2A.Z nucleosomes are also arranged in *Drosophila* genome in a manner of marking exon-intron structure. H2A.Z nucleosome occupancy is anticorrelated with genome sequence variability at TSS, TTS, and splicing sites. Genome sequence variability is high in the promoters and low in coding regions. Nucleosome occupancy around TSS is opposite. In the regions flanking splicing sites, both substitution and indel occurs more frequently in intons than in exons. Contrary to this, nucleosomes are enriched in exons and depleted in introns. However, we did not observe the rate heterogeneity between the linkers and H2A.Z nucleosomal DNA. Further analyses indicate that high sequence variability in the promoters and introns is likely attributed to high mutation rate in the enriched TRs and low functional constraint in introns, respectively.

The difference in genome sequence variability could be the result of different mutation rate and DNA repair efficiency. Previous studies on nucleosome-related rate heterogeneity concluded that nucleosome occupancy may increase mutation rate or inhibit DNA repair [19], [20], [21], [22]. In contrast, we did not observe the nucleosome-related rate heterogeneity in our study. This indicates that H2A.Z nucleosome occupancy may not increase mutation rate or inhibit DNA repair. To test this hypothesis, we obtained the classified old and young genes from the published work [28]. Briefly, any gene with an ortholog in bacteria was defined as an old gene, otherwise a young gene. Young genes evolve faster than old genes and change more frequently. Thus, it is sensitive to detect rate heterogeneity caused by nucleosomes on young genes. However, the correlation analysis of sequence variability between the linkers and nucleosomal DNA on young genes shows no rate heterogeneity (Fig. S7). No rate heterogeneity was observed in the alternative old/young gene sets defined by the presence/absence of a BLAST hit in *S. cerevisiae, D. hansenii, S. pombe*, and *C. neoformans* or *C. albicans* (data not shown). This indicates that H2A.Z nucleosome occupancy does not increase mutation rate in *Drosophila* genome.

**Figure 5:**
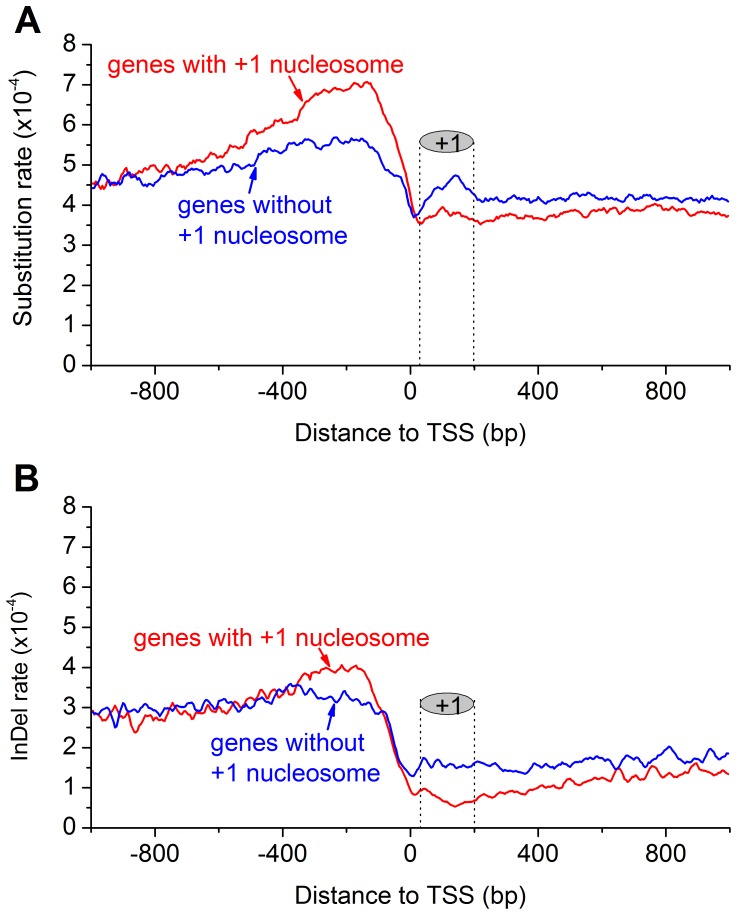
Genetic variation profiles around TSS of genes with and without +1 H2A.Z nuceosome. Both substitution (A) and indel (B) occur more frequently in the promoter regions than in genic regions downstream of TSS regardless of presence of +1 nucleosome. However, genomic sequence variability is lower in the genic regions where +1 nuclesomes reside than in the corresponding regions of genes without +1 nucleosome. The location of +1 nucleosome is indicated.

There are five major DNA repair pathways responsible for recognizing and repairing DNA damages in eukaryotic cells [29]. DNA repair event requires the assembly of DNA repair machinery at the sites of DNA damage composed of a large number of proteins [30], [31]. Nucleosome could form a barrier for DNA repair proteins, which leads to slow repair of nucleosomal DNA [32], [33]. RNA polymerase II (Pol II) stalled at DNA damage sites often resulted in lower repair efficiencies in occupied DNA [34]. Our previous study found that +1 H2A.Z nucleosome engaged in RNA Pol II pausing in *Drosophila* gene transcription [16]. To test whether H2A.Z nucleosome occupancy inhibits DNA repair, we classified genes into two groups: with or without +1 nucleosome. We further examined the sequence variability in the regions downstream of TSS where +1 nucleosomes reside. Interestingly, sequence variation rate in these regions is lower in genes with +1 nucleosome than in genes without +1 nucleosome (Fig. 5). Namely, the DNA repair efficiency is higher and/or mutation rate is lower in the +1 nucleosomal DNA sequences than in the naked linker regions. This suggests that presence of +1 nucleosome does not inhibit DNA repair or increase mutation rate. However, the mechanism of the interplay between +1 nucleosome occupancy and sequence mutation is unclear and needs further study. Notably, the sequence variability is also different in the promoter regions between these two groups of genes. Interestingly, the difference pattern is opposite between the promoter regions and the +1 nucleosome regions. We examined the enrichment of TRs in these two gene groups and found no significant difference. Therefore, other functional constraints may contribute to the complicated pattern of sequence variability involving nucleosome occupancy.

Nucleosome-related rate heterogeneity was reported in other organisms (yeast, medaka, human) [19], [20], [21], and missing in our study of H2A.Z nucleosome in *Drosophila*. The H2A.Z nucleosomes are enriched at gene starts and involve in transcription, for example, RNA Pol II pausing [16]. H2A.Z nucleosomes direct start site selection and initiate transcription in yeast in a different way, compared with *Drosophila* [3]. The transcription-coupled DNA repair may contribute to the different effect of nucleosome occupancy on sequence mutation among different species. Thus, the absence of rate heterogeneity in *Drosophila* may be in part due to the complex interplay between nucleosomes and gene transcription. As a matter of fact, our results imply that H2A.Z nucleosome occupancy has no impact on genome sequence variation in *Drosophila* genome, which is largely determined by sequence composition and the concomitant selection pressure.

## Supporting Information

Figure S1 **Indel rate around TSSs. Both 1 bp and >1 bp indel rates are higher in the promoter region than in the coding regions.** The inset specifically shows this rate heterogeneity of 1****bp indels.
(PNG)

Figure S2 **Profiles of sequence variations and nucleosome occupancy around splicing sites classified by exon size.** The exon size of the top, middle, and bottom panel is 100–200****bp, 250–300****bp, 450–500****bp, respectively. The profiles are independent of exon size. Substitution and indel rates are largely higher inside introns than in exons. The nucleosome occupancy is opposite. The number of nucleosome formed in each exon category is drawn at the top of its panel.
(PNG)

Figure S3 **Frequency of internal exon length in** ***Drosophila*** **genome.** Most exons are approximately 150 bp long, roughly the length of nucleosomal DNA.
(PNG)

Figure S4 **No rate heterogeneity between nucleosomal DNA and linker DNA of phased and delocalized nucleosomes.** There is no significant difference in substitution (A) and indel (B) rate between linkers and nucleosomal DNA. The vertical dotted lines indicate the border line between linkers and nucleosomes.
(PNG)

Figure S5 **Rate heterogeneity between boundaries and inside of nucleosomes spanning splicing sites.** The substitution (A) and indel (B) rates increase starting at 40 bp to the dyad and toward the nucleosomal border within intronic site. Only nucleosomes whose midpoints locate within exons but 40–60 bp internal to the splicing site are used in this plot. The vertical dotted lines indicate the border line between linkers and nucleosomes.
(PNG)

Figure S6 **Frequency of genomic sequence variation and tandem repeats (TRs) around TSS.** TRs are enriched in the promoter regions and peak at around 200 upstream of TSS where substitution and indel peak.
(PNG)

Figure S7 **No rate heterogeneity between nucleosomal DNA and linker regions residing in old and young genes.** There is no significant difference in substitution (A) and indel (B) rate between linkers and nucleosomal DNA. The vertical dotted lines indicate the border line between linkers and nucleosomes.
(PNG)
