# Supplementary figures and images for: H2A.Z Nucleosome Positioning Has No Impact on Genetic Variation in Drosophila Genome
Source: PLoS One. 2013 Mar 5;8(3):e58295. doi: 10.1371/journal.pone.0058295 (PMC3589275; doi:10.1371/journal.pone.0058295)

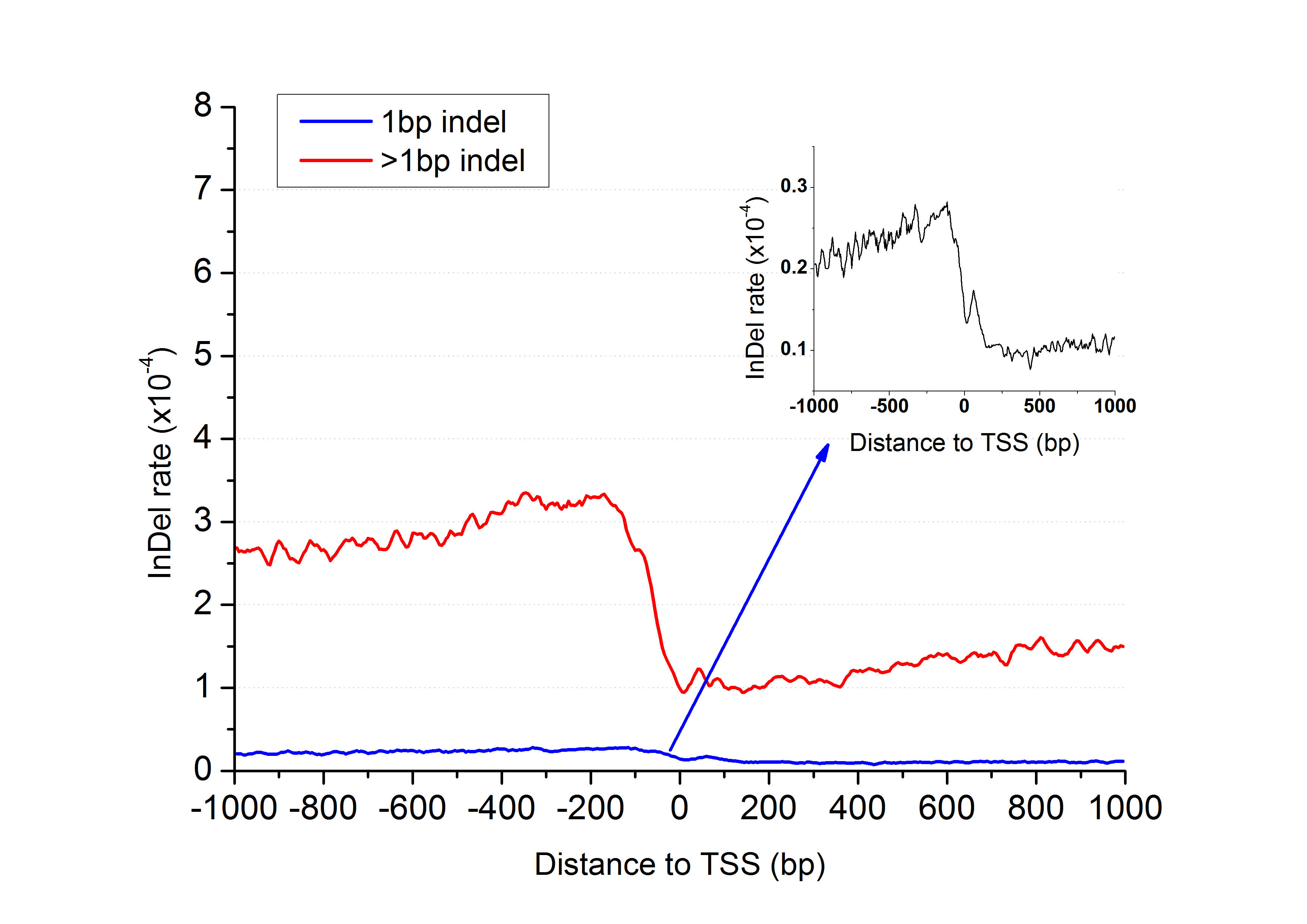

Supplement: Figure S1 — Indel rate around TSSs. Both 1 bp and >1 bp indel rates are higher in the promoter region than in the coding regions. The inset specifically shows this rate heterogeneity of 1bp indels. (PNG) [file pone.0058295.s001.png]

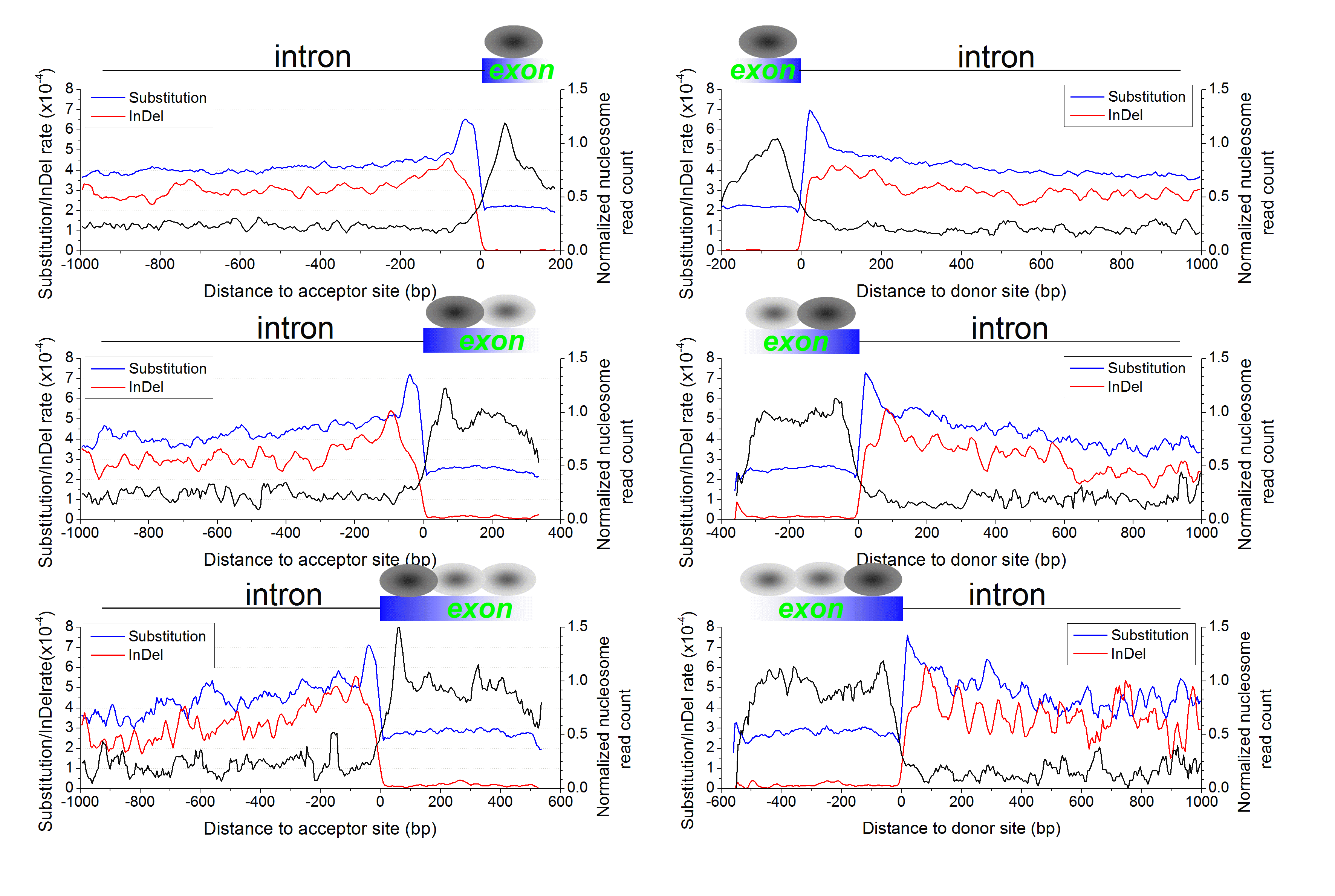

Supplement: Figure S2 — Profiles of sequence variations and nucleosome occupancy around splicing sites classified by exon size. The exon size of the top, middle, and bottom panel is 100–200bp, 250–300bp, 450–500bp, respectively. The profiles are independent of exon size. Substitution and indel rates are largely higher inside introns than in exons. The nucleosome occupancy is opposite. The number of nucleosome formed in each exon category is drawn at the top of its panel. (PNG) [file pone.0058295.s002.png]

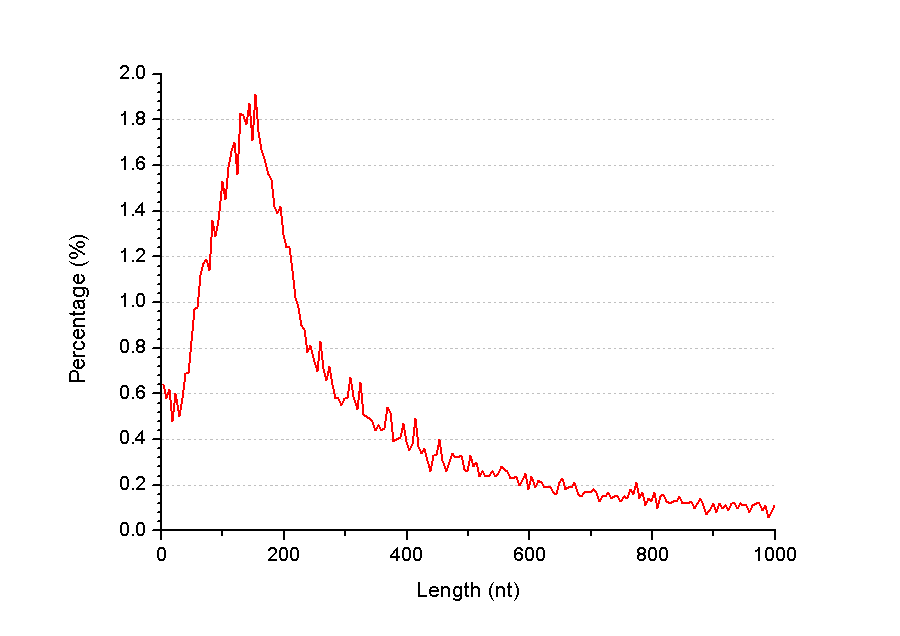

Supplement: Figure S3 — Frequency of internal exon length in Drosophila genome. Most exons are approximately 150 bp long, roughly the length of nucleosomal DNA. (PNG) [file pone.0058295.s003.png]

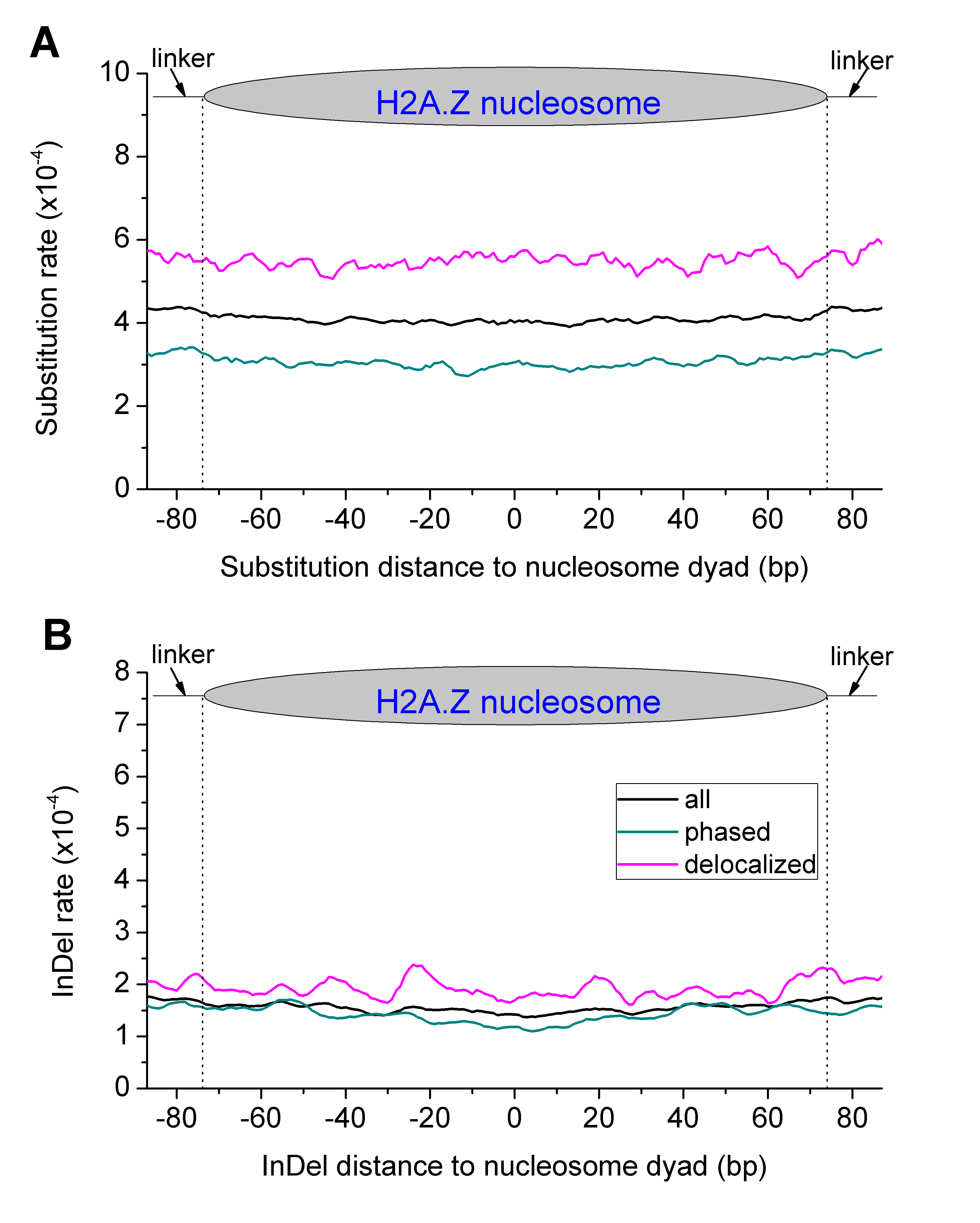

Supplement: Figure S4 — No rate heterogeneity between nucleosomal DNA and linker DNA of phased and delocalized nucleosomes. There is no significant difference in substitution (A) and indel (B) rate between linkers and nucleosomal DNA. The vertical dotted lines indicate the border line between linkers and nucleosomes. (PNG) [file pone.0058295.s004.png]

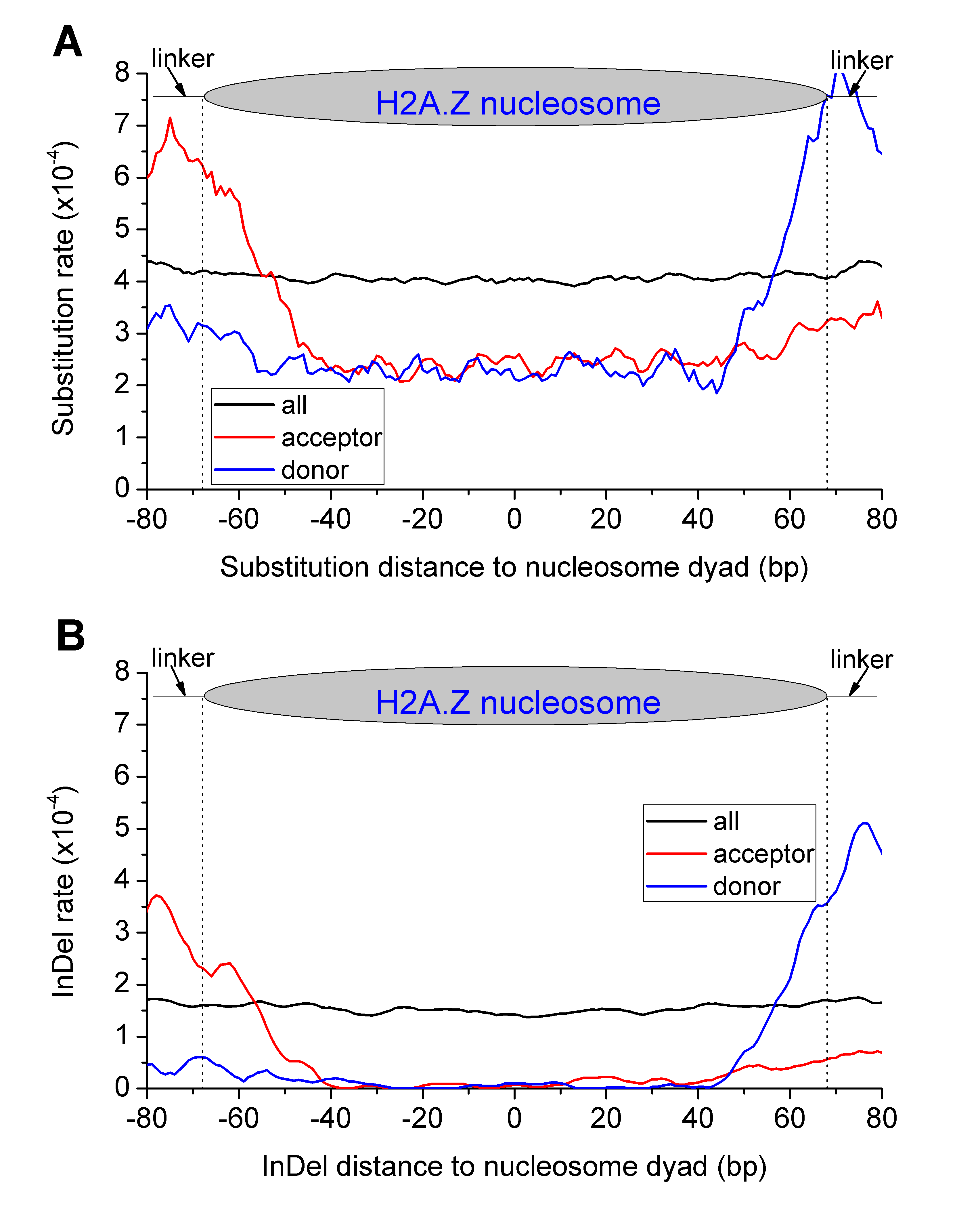

Supplement: Figure S5 — Rate heterogeneity between boundaries and inside of nucleosomes spanning splicing sites. The substitution (A) and indel (B) rates increase starting at 40 bp to the dyad and toward the nucleosomal border within intronic site. Only nucleosomes whose midpoints locate within exons but 40–60 bp internal to the splicing site are used in this plot. The vertical dotted lines indicate the border line between linkers and nucleosomes. (PNG) [file pone.0058295.s005.png]

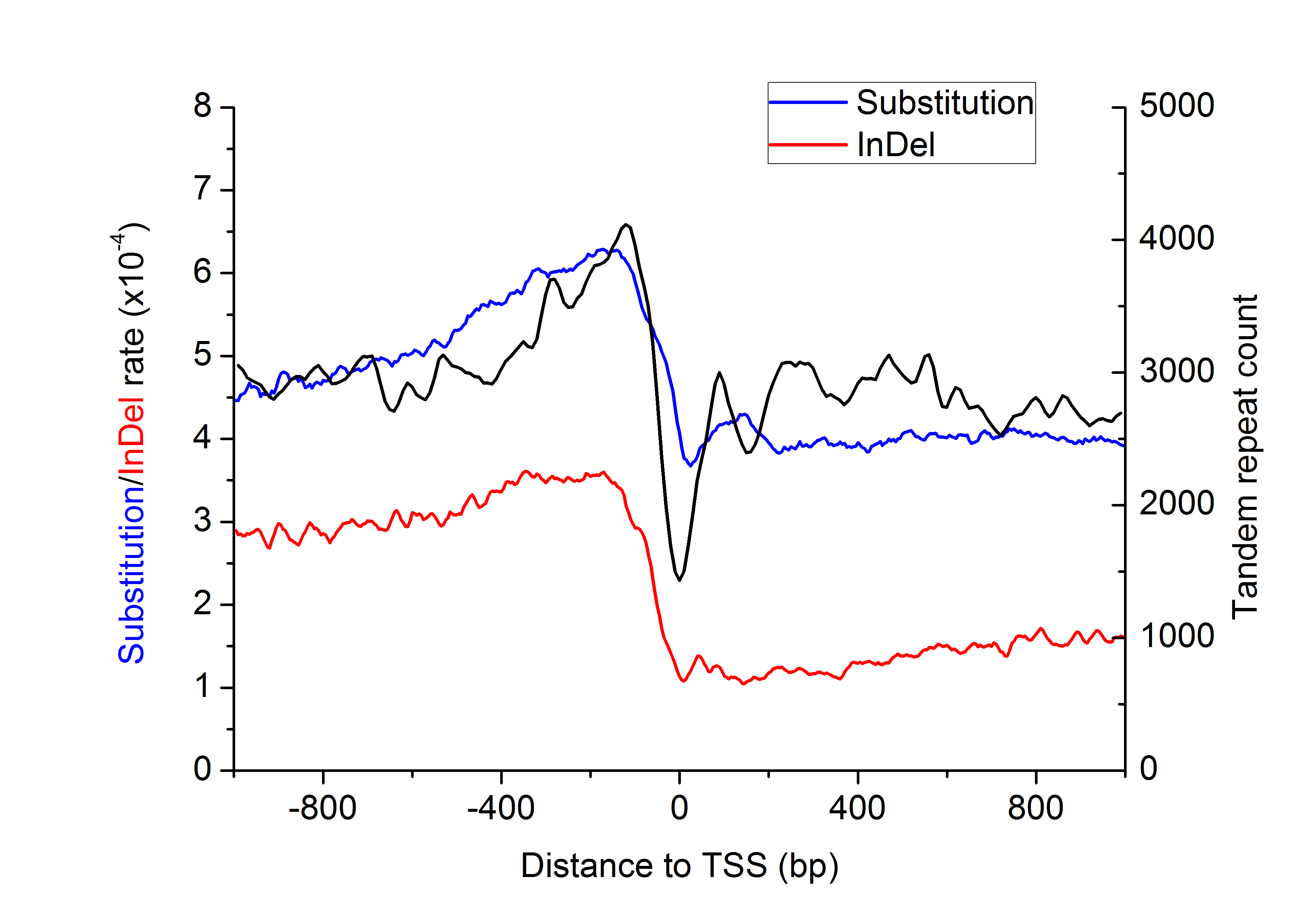

Supplement: Figure S6 — Frequency of genomic sequence variation and tandem repeats (TRs) around TSS. TRs are enriched in the promoter regions and peak at around 200 upstream of TSS where substitution and indel peak. (PNG) [file pone.0058295.s006.png]

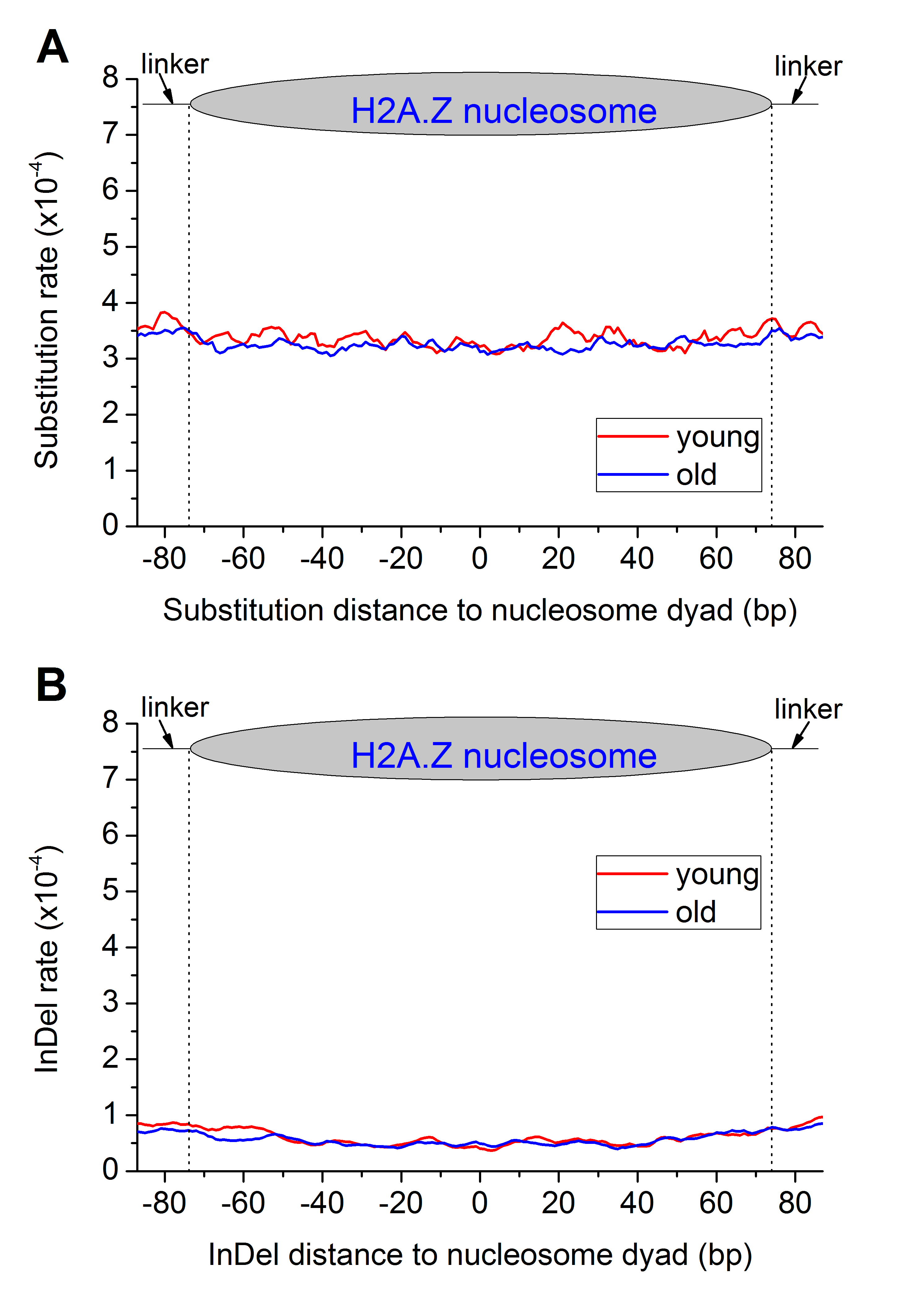

Supplement: Figure S7 — No rate heterogeneity between nucleosomal DNA and linker regions residing in old and young genes. There is no significant difference in substitution (A) and indel (B) rate between linkers and nucleosomal DNA. The vertical dotted lines indicate the border line between linkers and nucleosomes. (PNG) [file pone.0058295.s007.png]
